# Whole genome sequencing data of *Chromobacterium amazonense* BASUSDA_45 isolated from soil in Bangladesh capable of degrading pesticide

**DOI:** 10.1016/j.dib.2022.108853

**Published:** 2022-12-24

**Authors:** Md Atikur Rahman, Khaled Mahmud Sujon, Mohammad Tanbir Habib, Md. Forhad Hossain, K.M.K.B. Ferdaus, K.M.F. Hoque, Zennat Ferdousi, Md Abu Reza

**Affiliations:** a Department of Genetic Engineering and Biotechnology, Molecular Biology and Protein Science Laboratory (MBPSL), University of Rajshahi, Rajshahi 6205, Bangladesh; b Bangladesh Sericulture Development Board, Rajshahi, Bangladesh; c Institute for Developing Science and Health Initiatives (ideSHi), 167/24, Blue moon Tower, 11th Floor ECB Chattar, Shagufta New Rd, Dhaka 1216, Bangladesh

**Keywords:** WGS, Pesticide, Cypermethrin, Microbial genomics, Bioinformatics, Sequence assembly, Biodegradation, PGAP

## Abstract

This article reports the *Chromobacterium amazonense* BASUSDA_45 strain's draft genomic sequence. The bacterium was isolated from cypermethrin pesticide contaminated soil and then the sequencing was carried out. Initially *de novo* assembly of the raw sequences, trimming and quality check generates 125 contigs having N50 of 78,923. Further mapping of the contigs generated scaffolds. The genome contains 53 scaffolds with a total length of 4,295,151 bp having 62.30% GC content and N50 of 3,726,017. Annotation using Prokaryotic Genome Annotation Pipeline (PGAP) reveals 4181 genes among which 4096 were coding sequences, 76 tRNAs, 3 rRNAs, 4 noncoding RNAs. The raw sequence reads and annotated genome were uploaded to NCBI's Bioproject repository with the accession number PRJNA686506.

**Specification Table**

**Table:** 

Subject	Agricultural Sciences
Specific subject area	Agricultural Microbiology
Type of data	Assembled and annotated draft genome of *C. amazonense* strain BASUSDA_45
How the data were acquired	Illumina DNA Prep – (M) Tagmentation (96 Samples) library preparation kit (catalog number 20018705; San Diego, CA, USA), Illumina MiSeq, FastQC v0.11.19, Trimmomatic v0.39, SPAdes v3.14.1, RagTag v1.0.1, PlasmidFinder v2.0, NCBI Prokaryotic Genome Annotation Pipeline (PGAP)
Data format	Raw
Description of data collection	DNA extraction was performed from pure culture by TIANamp bacteria DNA kit (Tiangen Biotech Co. Ltd., Beijing, China) according to the manufacturer's protocol. 151-bp paired-end sequencing genomic library was constructed using the Illumina DNA Prep – (M) Tagmentation (96 Samples) library preparation kit(catalog number 20018705; San Diego, CA, USA) following the manufacturer's protocol. Sequencing was carried out in Illumina MiSeq platform. The raw sequence reads were used for genome assembly and subsequently for annotation.
Data source location	Institution: Molecular Biology and Protein Science Laboratory (MBPSL) City/Town/Region: Rajshahi, Country: Bangladesh Latitude/longitude for collected sample: 24.87947 N 90.70843 E
Data accessibility	The data is stored in the public repository, NCBI. Bioproject: PRJNA686506 NCBI BioSample: SAMN17119081 NCBI GenBank Accession Number: JAFCYX000000000.1 Assembly: GCA_016858235.1

## Value of the Data

• The strain is capable in degrading pesticide (cypermethrin) supplemented into the media as the sole carbon source for their growth, development and metabolism. The mechanism is yet to be revealed how this bacterium use and utilize pesticide for their survival. Following utilization, this bacterium greatly degrades pesticide, reducing toxic waste and can be an eco-friendly measure to help protecting the environment.
• The reported data for the *C. amazonense* strain BASUSDA_45 is very significant for the both fundamental and applied microbial research as a means of eco-friendly environmental control.
• The sequencing data can be further analyzed to find particular gene that degrades pesticides and other genes that utilize pesticide residues as energy source. Again, particular pathways that involve these processes can give detail insights.
• Researchers that are involved in and finding ways to reduce toxic chemicals from environment can be highly benefitted from these data.

## 1 Data Description

*Chromobacterium amazonense* is a Gram negative bacterium mostly found in soil and water [1]. They are free-living bacteria able to survive under different adverse environmental conditions [2]. *C. amazonense* strain BASUSDA_45 was isolated from pesticide contaminated soil. We reported the whole genome sequencing data of *Chromobacterium amazonense* strain BASUSDA_45, here.

## 2 Experimental Design, Materials and Methods

1. Source of Inoculum and Bacterial Culture

The sample was collected from pesticide contaminated soil of Netrokona, Bangladesh (24.87947N 90.70843 E) where pesticides had been continuously used for at least 10years. The soil was collected at a depth of 25 cm from the surface and serial dilution was carried out until adequate colonies are obtained. From that, pure culture was done and DNA extraction was performed.

2. DNA Extraction

The extraction of DNA was carried out by TIANamp bacteria DNA kit (Tiangen Biotech Co. Ltd., Beijing, China) following the manufacturer's protocol. From these onward downstream processes were conducted by sequencing service provider Invent Technologies Ltd. (Dhaka, Bangladesh).

3. Library Preparation

151-bp paired-end sequencing genomic library was constructed using the Illumina DNA Prep – (M) Tagmentation (96 Samples) library preparation kit (catalog number 20018705; San Diego, CA, USA) according to the manufacturer's protocol. Illumina MiSeq platform was used to sequence the prepared library.

4. Whole Genome Sequencing and Data Analysis

Illumina Miseq platform yielded raw sequence reads exceeding 52x coverage. The quality of the raw sequence reads was assessed using FastQC v0.11.9 (https://www.bioinformatics.babraham.ac.uk/projects/fastqc). Trimmomatic v0.39 was used to remove the adapter sequences using ILLUMINACLIP:adapters/NexteraPE-PE.fa:2:30:10:2:keepBothReads and quality was ensured using the LEADING:3 TRAILING:3 MINLEN:36 options (http://www.usadellab.org/cms/?page=trimmomatic) [3]. De novo assembly with SPAdes v3.14.1 (https://cab.spbu.ru/software/spades) produced 157 contigs with a N50 of 118989 bp [4]. The coverage threshold was computed automatically using "—cov-cutoff auto" for the SPAdes assembly, and the k-mer sizes were set to "-k 21,33,55,77," with the "—careful" option used to cut the levels of mismatches in the finally produced contigs. The assembled data was mapped to the reference genome *Chromobacterium paludis* strain IIBBL 257-1 (GenBank accession number CP043473.1) by using the scaffolding software RagTag v1.0.1 (https://github.com/malonge/RagTag), yielding 167.84Ns per 100 kbp and 114 scaffolds, with a N50 value of 3726,017 bp [5]. Unless otherwise indicated, default parameters were utilized for all applications. The NCBI Prokaryotic Genome Annotation Pipeline was used to annotate the draft genome (PGAP) [6,7]. The genome assembly size of *Chromobacterium amazonense* strain BASUSDA_45 was 4295,151 bp distributed in 53 scaffolds above 200 bp and representing 4287,951 bp of ungapped sequences (GC content, 62.30%), encompassing 4181 total genes, 76 tRNAs, 3 rRNAs, 4 noncoding RNAs.

## Ethics Statements

The permission was obtained from Institute of Biological Sciences, University of Rajshahi for the sampling of the soil microbes. This was approved in the *“Resolution No. of the 71th meeting of the Board of Governors of the Institute of Biological Sciences and Resolution No. 57 of the meeting of the Syndicate of the University of Rajshahi, Bangladesh.”*

## CRediT authorship contribution statement

**Md Atikur Rahman:** Conceptualization, Methodology, Data curation, Investigation, Writing – original draft. **Khaled Mahmud Sujon:** Data curation, Writing – original draft, Software, Formal analysis. **Mohammad Tanbir Habib:** Software, Formal analysis, Visualization, Investigation. **Md. Forhad Hossain:** Methodology, Investigation, Data curation. **K.M.K.B. Ferdaus:** Project administration. **K.M.F. Hoque:** Supervision, Project administration. **Zennat Ferdousi:** Project administration. **Md Abu Reza:** Supervision, Resources, Project administration, Funding acquisition, Writing – review & editing.

## Declaration of Competing Interest

The authors declare that they have no known competing financial interests or personal relationships that could have appeared to influence the work reported in this paper.

## Data Availability

Chromobacterium amazonense strain:BASUSDA_45 Genome sequencing and assembly (Original data) (NCBI)
